# The value of immunohistochemistry in sentinel lymph node histopathology in breast cancer

**DOI:** 10.1038/sj.bjc.6602641

**Published:** 2005-06-07

**Authors:** M B Klevesath, L G Bobrow, S E Pinder, A D Purushotham

**Affiliations:** 1 Cambridge Breast Unit, Department of Surgery, Addenbrooke's Hospital, Cambridge University Hospitals NHS Foundation Trust, Cambridge, England, UK; 2 Department of Pathology, Addenbrooke's Hospital, Cambridge University Hospitals NHS Foundation Trust, Cambridge, England, UK

**Keywords:** breast neoplasms/pathology, lymphatic metastasis/pathology, sentinel lymph node biopsy/methods, immunohistochemistry

## Abstract

The optimal protocol for the histopathological examination of sentinel lymph nodes (SLNs) in breast cancer has not been determined. The value of more detailed examination using immunohistochemistry (IHC) is controversial. A total of 476 SLNs from 216 patients were reviewed. Sentinel lymph nodes were sectioned at three levels at 100 *μ*m intervals and stained with haematoxylin and eosin (H&E). If the H&E sections showed no evidence of metastasis, then the three serial sections were stained with a murine monoclonal anti-cytokeratin antibody (CAM 5.2). Metastatic deposits were classified as macrometastasis (>2.0 mm), micrometastasis (0.2–2.0 mm) or isolated tumour cells (ITC, <0.2 mm). Of the 216 patients, 56 (26%) had metastasis as identified by H&E. Immunohistochemistry detected metastatic deposits in a further nine patients (4%), of whom four (2%) had micrometastasis and five (2%) had ITC only. Those cases with micrometastases were all, on review, visible on the H&E sections. Immunohistochemistry detects only a small proportion of metastasis in SLNs. All metastatic deposits identified by IHC were either micrometastasis or ITC. Until the prognostic significance of these deposits has been determined, IHC may be of limited value in the histopathological examination of SLNs.

Breast carcinoma is the leading cause of death in women with over 300 000 deaths annually worldwide (Pisani *et al*, 1999a, 1999b). Axillary lymph node (ALN) status is one of the most important prognostic factors in breast cancer. Traditionally, ALN staging has been achieved by histopathological examination of lymph nodes retrieved during ALN dissection (ALND) or four-node sampling of the axilla. Modern standard histopathological work-up in most institutions consists of haematoxylin & eosin (H&E) staining of a limited number of macroscopic slices of the lymph node, usually between 1 and 4, depending on the size of the node. This may underestimate the disease status in some patients. Recent studies have shown that more exhaustive examination of the lymph nodes by serial sectioning, immunohistochemistry (IHC) or molecular techniques (e.g. by polymerase chain reaction, PCR) increases the detection of ALN metastasis compared with routine H&E methodology (van Diest *et al*, 1999; Cserni, 2004). The major disadvantage of these techniques is that they are highly labour-intensive and too time-consuming to be routinely applied to all lymph nodes retrieved in an ALND. Secondly, the evidence that axillary node status is of prognostic significance is based on routine H&E methodology, often from historical series in which a smaller proportion of the lymph node was examined than is standard practice today.

More recently, sentinel lymph node biopsy (SLNB) has been proposed as an alternative method for staging the axilla in women who have early breast cancer with clinically node negative axillae (Giuliano *et al*, 1994; Veronesi *et al*, 1997; McIntosh and Purushotham, 1998; Veronesi *et al*, 1999; Veronesi *et al*, 2003). The SLN is the first lymph node to receive lymphatic drainage from a tumour. It is therefore the node most likely to contain metastatic breast carcinoma. A tumour-free SLN virtually excludes lymphatic involvement of the entire regional lymphatic basin (Turner *et al*, 1997). Sentinel lymph node biopsy allows a more detailed histological analysis to be performed within the context of a routine histopathology laboratory, as more sections can be scrutinised and additional techniques such as immunohistochemical staining methods can be applied, if desired. However, there is currently no internationally accepted standardised protocol for the histological examination of SLNs. As a result, some institutions have developed their own, in-house guidelines, and many use a combination of serial sectioning and/or IHC.

We carried out a retrospective review of the histopathological assessment of SLNs in our unit with the aim of determining the value of IHC in the detection of metastatic deposits in SLNs when compared to H&E examination alone.

## PATIENTS AND METHODS

### SLN biopsy

Between November 1999 and September 2004, 216 patients underwent SLNB at Addenbrooke's Hospital in Cambridge, UK. Written informed consent was obtained from all patients. Patients considered eligible for SLN had unifocal tumours up to 40 mm on ultrasound, with a proven histopathological diagnosis of invasive breast cancer on core biopsy. Pregnant patients, patients with clinically involved axillary nodes or with multifocal breast cancer, previous diagnostic excision biopsy and those who previously had treatment for breast cancer (e.g. neoadjuvant chemotherapy) were excluded from the study.

All patients underwent conventional wide local excision (WLE) or mastectomy to remove the primary tumour. The SLN was detected using a dual technique of radioisotope and patent blue dye. Briefly, between 2 and 24 h prior to the operation, a single dose of up to 40 MBq of 99mTc-nanocolloid (0.2 ml; Gipharma S.r.l., Italy) was injected into the breast. Routes for injection included the subdermal, intradermal and intratumoral route. Subdermal/intradermal injections were performed either around the areola or into the skin overlying the tumour. After induction of anaesthesia, a dose of 2.0 ml patent blue dye (2.5% Bleu patenté V®, Guerbet, France) with 3.0 ml 0.9% saline was injected in the periareolar region of the breast. The area was massaged for 5 min to optimise uptake of the dye by the lymphatics. The SLN was identified by its bluish discolouration. If no blue node was detected, a gamma probe was used to trace the SLN. The node(s) in question were excised and the wound explored with the probe for additional blue and/or ‘hot’ nodes that might represent further SLNs. If the SLNs revealed histological evidence of metastatic spread (i.e. macrometastasis or micrometastasis), the patient was readmitted for an ALND. If only isolated tumour cells (ITC) were detected in the SLNs, a further surgical procedure was carried out only in exceptional circumstances.

### Histopathology of the SLNs and ALNs

All SLNs measuring less than 5 mm in maximum diameter were bisected and both halves processed for histological examination. Nodes, which were greater than 5 mm in maximum diameter, were sliced into at least three slices at approximately 2–3 mm intervals and all the slices were embedded, in as many cassettes as were required. All slices of each node were routinely processed through to paraffin wax. Blocks were sectioned at three levels at 100 *μ*m intervals and stained with H&E. If the H&E sections showed no evidence of metastasis on histological examination, then the three serial sections from all blocks were stained with a murine monoclonal anti-cytokeratin antibody (CAM 5.2, Becton-Dickinson Biosciences, UK).

The lymph nodes removed during conventional axillary dissection were examined according to National Health Service Breast Screening Programme (NHSBSP) pathology reporting guidelines (NHS Cancer Screening Programmes & The Royal College of Pathologists, 2005). Briefly, nodes greater than 5 mm in diameter were sliced at 2–3 mm intervals and several slices examined in a single cassette, those less than or equal to 5 mm in diameter were embedded unsliced. Sections from each lymph node were stained with H&E. Immunohistochemistry was not performed on lymph nodes obtained from standard ALND, except where required by the histopathologist for investigation of suspicious cells.

For histological reporting of SLNB and ALND specimens, UK National Guidelines were used (NHS Cancer Screening Programmes and The Royal College of Pathologists, 2005). Specifically, all metastases greater than 2.0 mm in size were classified as macrometastasis. A micrometastasis was diagnosed when one or more deposits of metastatic carcinoma were seen measuring more than 0.2 mm in size, but none of which was larger than 2.0 mm. Isolated tumour cells were reported when single or small clusters of tumour cells were identified measuring not more than 0.2 mm in maximum dimension.

## RESULTS

**Figure 1:**
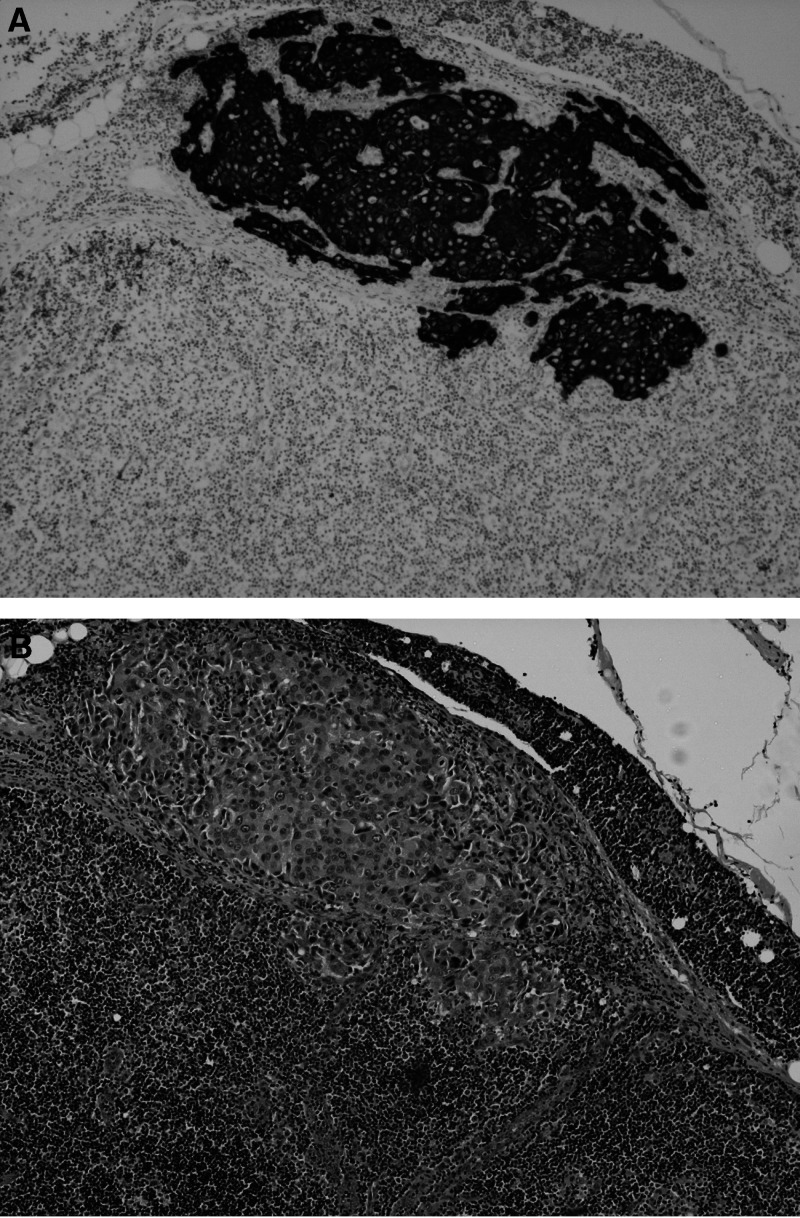
A micrometastatic deposit present only in the levels of this SLN shown by IHC (**A**) and on H&E (**B**).

**Figure 2:**
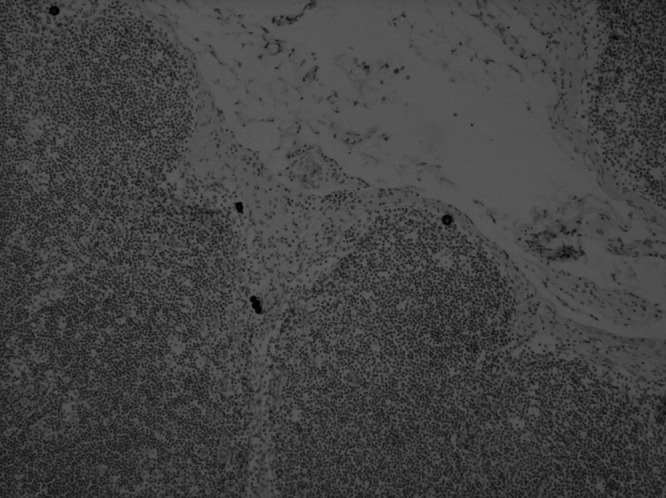
ITCS present in the periphery of this SLN were only identified with IHC and were not even on review, visible on H&E.

The histopathological characteristics of the primary tumours are shown in Table 1. A total of 476 SLNs were available for analysis (mean 2.2 per patient, range 1–7). Of the 216 patients analysed, 56 (26%) had SLN metastasis as identified by H&E only. A further nine patients (4%) had metastatic deposits detected by IHC (Table 2), of whom four (2% of total, 44% of those detected by IHC) had micrometastatic disease (Figure 1), ranging from 0.2 to 1.0 mm in size. The remaining five patients had ITC (3% of total patients, 56% of those detected by IHC; Figure 2). One patient (A) had a suspicious 0.2 mm deposit identified on H&E, and an actin immunohistochemical stain was performed to determine the presence or absence of a myoepithelial layer. The micrometastases were readily identified on the original H&E sections in all four cases when the slides were reviewed (by SEP). In no case were ITC detected when the original H&E slides were reviewed.

Seven of the IHC positive patients had an ALND. In all seven patients, SLN was the only positive node in this series.

## DISCUSSION

Sentinel lymph node biopsy is rapidly emerging as an alternative to ALND in staging the axilla in patients with early breast cancer and is routinely performed in many institutions around the world. Although SLNB has recently been incorporated into the TNM classification (Sobin *et al*, 2002), the optimal histopathological workup of SLNs is currently not standardised. A recent survey of practices in a large number of European institutions revealed wide discrepancies internationally as well as nationally (Cserni *et al*, 2004a). Although some countries have set up national guidelines for specimen handling, many institutions have developed their own guidelines for SLN processing, which are more intensive than the national guidelines recommend as a minimum, and which are frequently determined by the institution's research strategy.

The issue is further complicated by the rapid advances in molecular techniques that allow identification of even the smallest metastatic deposits down to single isolated tumour cells. Almost all data relating to the prognostic significance of ALN involvement in invasive breast cancer are based on examination of a single standard H&E-stained section, often of one slice of each lymph node. Invariably, the majority of the metastases detected by this technique would have been what would now be classified as macrometastasis.

Two issues need to be taken into consideration when designing a protocol for SLN examination. Firstly, what is the minimum size of metastatic deposit that one should aim to identify (i.e. macrometastasis, micrometastasis or ITC)? Secondly, what is the optimal protocol for detecting a metastatic deposit of a given size? There are as yet no definitive answers to these issues, although some evidence is available that may offer some guidance as to how to address these issues.

It is self evident that a more detailed examination of lymph nodes increases the percentage of metastases found (Sapir and Amromin, 1948; Pickren, 1961). Recent reports have estimated that step-sectioning with H&E staining and IHC results in the ‘upstaging’ of about 10 and 20% of patients, respectively (van Diest *et al*, 1999; Cserni, 2004). The majority of these tumour deposits are likely to be micrometastasis or ITC.

The prognostic significance of SLN micrometastasis, whether detected by H&E serial sectioning, IHC or a combination of both is controversial (Dowlatshahi *et al*, 1997; Tjan-Heijnen *et al*, 2001; Noguchi, 2002). The contradictory reports in the literature probably reflect the heterogeneity of this population of tumours in terms of their metastatic potential. The on-going American College of Surgeons Oncology Group (ACOSOG) Z0011 trial will help to determine the significance of SLN micrometastasis detected by IHC (protocol available at ACOSOG) (www.acosog.org).

Until further evidence regarding the prognostic significance of SLN micrometastasis is available, it would seem reasonable to propose that the identification of macrometastases should be the minimum standard in SLN histopathology. A number of recent reports have applied mathematical models of SLN metastasis in an attempt to determine the optimal histopathological protocol that allows a metastasis of a defined size to be identified (Meyer, 1998; Farshid *et al*, 2000; Cserni, 2004). Cserni (2004) demonstrated that a step-sectioning protocol of levels separated by 250 *μ*m will detect all macrometastases of a size of 2.0 mm or greater. However, if a higher threshold for the detection of macrometastases is chosen, the workload can be reduced considerably. For example, by examining levels separated by 1.0 mm, all macrometastases measuring 2.2 mm or more will be detected, resulting in a reduction of the workload by 75%. Since metastases of this size are usually seen on H&E stains, IHC will only be required in rare cases where the epithelial nature of suspicious cells needs to be confirmed.

If the aim of the histopathological examination of the SLN is to identify micrometastases as well, then the extent of the protocol has to be increased considerably. On the basis of the geometrical model described by Cserni (2004), sections separated by 200 *μ*m would detect all micrometastases and not misclassify them as ITC. These sectioning protocols require that the entire node is sectioned, resulting in a considerable workload for the pathology department.

Farshid *et al* (2000) developed a mathematical model of an average SLN from histological survey data. In a series of simulations, the virtual SLN generated by the model was subjected to various sectioning protocols currently in use that were evaluated for their ability to detect micrometastatic deposits of specified sizes. In addition, the authors performed a cost analysis for the different protocols. Slicing the node into fine slices as opposed to bisecting it increased the chances of detecting micrometastasis in the range of 50 *μ*m to 2.0 mm. The increase was two-fold for 2 mm slices and four-fold for 1 mm slices. The increased detection rate could be achieved at a small increase in cost. However, the costs for this approach rose significantly when applied to larger lymph nodes, as in these cases the tissue usually had to be embedded in several blocks. Consistent with other reports, the inclusion of serial sections increased the likelihood of detecting micrometastasis. Again, only the most detailed protocol consisting of serial sections of fine slices of the entire node was able to detect all micrometastasis of ⩾200 *μ*m. In all, only two of the six protocols evaluated had detection rates of 30% or higher for micrometastasis of ⩾500 *μ*m. Interestingly, cost was not a good discriminator for efficiency. Two of the better performing strategies were more expensive, but two other protocols with similar costs had poor detection rates. The value of using IHC on SLN sections was not addressed by the model.

It has been demonstrated that the majority of metastases will be detected in the first few sections examined (Cserni, 2002; Yared *et al*, 2002). Yared *et al* (2002) examined 10 levels of serially sectioned SLNs and performed IHC on levels 3 and 8, while the remaining levels were stained with H&E. The authors found that the first two H&E or the first cytokeratin-stained levels were positive for metastases in 96%. Two additional H&E-stained and one cytokeratin-stained level of each SLN correctly identified the status of the node in 98% of cases. It seems, therefore, that limiting the number of sections to the initial few, at intervals that will reliably identify macrometastases, might represent a reasonable compromise between sensitivity and workload.

An alternative strategy to limit the extent of the analysis would be to examine those areas of the SLN first that are most likely to contain metastatic deposits. Two recent studies have suggested that metastatic deposits have a higher probability of being located in the region of the inflow junction of the afferent lymphatic vessel (Cserni, 2000; Diaz *et al*, 2003). Theoretically, and speculatively, this region could potentially be marked by the surgeon intraoperatively when the blue-stained lymphatic is followed to the SLN, and sections with a higher probability of containing metastases could then be targeted at the initial histopathological examination. Further, more detailed evaluation would be carried out only if the initial sections were negative. In practice, however, this may be impossible and further evidence will be required to determine the value of this strategy.

If, as the evidence from the literature suggests, serial sectioning increases the detection rate of SLN metastasis, what is the place of IHC? Immunohistochemistry facilitates detection of small metastatic deposits by direct labelling of the tumour cells. Review of slides stained by IHC would require less time by the pathologist, which in turn would allow a greater number of sections to be screened. On the other hand, increasing the number of immunohistochemically prepared sections will increase costs and put considerable strain on laboratory resources. As SLN biopsy becomes the standard of care in many institutions, IHC requests would lead to an exponential increase in costs and few laboratories will have the facilities or resources to meet the demand. As an extreme example, Weaver (2003) has calculated the costs for performing IHC on 10 *μ*m sections on an average of two SLNs per patient in the US. Leaving aside interpretive costs, this would amount to $4800 per case, or more than $690 million annually, and approximately 57 million slides would have to be screened per year. Apart from costing and resources issues, human error and false positive results need to be taken into account. Rescreening of IHC slides by an automated image analysis system revealed missed metastatic deposits in up to 10% of patients (Weaver *et al*, 2003). Furthermore, considerable interobserver variability in reading IHC-stained sections of SLN has been reported (Roberts *et al*, 2003). False positive results can occur with some of the most commonly employed low molecular weight cytokeratin antibodies (Domagala *et al*, 1992; Xu *et al*, 2000; Rao *et al*, 2005). To avoid false positivity caused by fibroblastic-type reticular cells and other cells, assessment needs to be based on immunoreactivity and morphological criteria. Thus, an H&E-stained section is also required. Potentially, if H&E and IHC are used in combination, the H&E slides may not be scrutinised as meticulously because the pathologist may rely on the subsequent IHC stain to pick up any missed metastases. This may have been the case in the present series, as the micrometastases identified by IHC were visible on H&E when the sections were reviewed. Careful examination of H&E sections at levels of 250 *μ*m is sufficient to identify the large majority of SLN macrometastases. A proportion of micrometastases will also be identified by this protocol but inevitably some will be missed. The main advantage of IHC lies in facilitating the identification of some additional very small (<2.0 mm) deposits and ITCs, although many micrometastases will be detected by careful scrutiny of the H&E sections alone. Current UK Pathology guidelines do not recommend the routine use of IHC on SLN outside clinical trials and aim to identify the macrometastatic disease (NHS Cancer Screening Programmes & The Royal College of Pathologists, 2005). We hope, with our strategy of undertaking additional levels, to identify some (but not all) micrometastatic disease, as described above. In our hands, IHC was of little additional value to the initial H&E stains. Over half of the deposits detected by IHC (five out of nine) were ITC alone and were not established metastatic deposits. Since the prognostic value of ITC is unknown and further therapeutic decisions are not based on the presence of ITC as a rule, omitting IHC would not have altered management in these patients. The remaining four IHC-positive cases, 2% of the entire study population, consisted of micrometastases, all of which were readily identified on the original H&E stains when the slides were reviewed. None of the micrometastases identified on IHC had further metastases in the non-SLN. Others have found non-SLN metastasis in approximately 9% of SLNs positive on IHC only (Cserni *et al*, 2004b). This difference may be due to the small number of cases observed in our study or due to differences in histological protocols.

As a result of this study, we have changed our practice and have abandoned IHC on SLN, unless suspicious cells on the initial H&E stain require further investigation. This has reduced our costs and turnaround time. This study did not address the value of step sectioning, but evidence from the literature suggests that if additional tests are to be performed on the SLN, this might be the most cost effective.

Results from the clinical trials looking at the prognostic significance of micrometastasis will help to determine whether techniques for detection of these smaller deposits should be included in routine SLN specimen handling protocols.

## Figures and Tables

**Table 1:** Histopathological characteristics of the study population

**Characteristic**	**No (%)**
*n*	216 (100)
*Mean tumour size in mm (range 4–40 mm)*	15.1
0–9	35 (16)
10–19	131 (61)
20–29	37 (17)
30–40	13 (6)
*Tumour type*	
Invasive ductal/NST	155 (72)
Invasive lobular	20 (9)
Special types	41 (19)
*Histological grade*	
1	46 (21)
2	110 (51)
3	60 (28)
*Receptor status*	
ER positive	189 (87)
ER negative	27 (13)
*Lympho-vascular invasion*	
Absent	190 (88)
Present	26 (12)

NST=no special type; ER=oestrogen receptor.

**Table 2:** Characteristics of SLN (micro)metastases missed on initial H&E examination

			**Tumour characteristics**					
**Patient ID**	**Size of metastasis (mm)**	**Seen on review of H&E**	**Size (mm)**	**G**	**T**	**LVI**	**ER**	**Non-SLN metastasis**
A	0.2	Yes	11	1	ST	No	+ve	No
B	0.6	Yes	20	2	ST	No	+ve	No
C	1.0	Yes	21	3	NST	Yes	−ve	No
D	0.9	Yes	23	2	NST	No	+ve	No
E	ITC	No	25	2	ILC	No	+ve	No
F	ITC	No	20	2	NST	No	+ve	No
G	ITC	No	13	2	NST	No	+ve	No
H	ITC	No	10	2	NST	Yes	+ve	N/A^a^
I	ITC	No	32	2	NST	No	+ve	N/A^a^

ITC=isolated tumour cells; H&E=haematoxylin and eosin; G=grade; T=tumour type; LVI=lympho-vascular invasion; ER=oestrogen receptor; SLN=sentinel lymph node; ILC=invasive lobular carcinoma; NST=invasive carcinoma of ductal/no special type; ST=invasive carcinoma of special type.

a ALND (axillary lymph node dissection) not done.
